# Evaluation of buffaloes’ follicular dynamics and stress state under different ovulation synchronization protocols

**DOI:** 10.21451/1984-3143-AR2018-0042

**Published:** 2018-08-16

**Authors:** Edmilson Daniel Stella, Alcides de Amorim Ramos, Felipe Rydygier de Ruediger, Ariane Dantas, Paulo Henrique Yamada, Viviane Maria Codognoto, Letícia Cristina Salgado, Suzane Brochine, Eunice Oba

**Affiliations:** 1 Departamento de Reprodução Animal e Radiologia Veterinária, Faculdade de Medicina Veterinária e Zootecnia, Universidade Estadual “Júlio de Mesquita Filho” (UNESP), Botucatu, SP, Brasil.; 2 Departamento de Produção Animal, Faculdade de Medicina Veterinária e Zootecnia, Universidade Estadual “Júlio de Mesquita Filho” (UNESP), Botucatu, SP, Brasil.

**Keywords:** cortisol, eCG, FTAI, hCG, progesterone

## Abstract

The aims of the present study were to analyze the effect of different hormonal protocols using an implant containing Norgestomet, at the ovulation synchronization in buffaloes and to verify the effect of the stress caused by manipulation of the herd during the experiment. Twenty-four female Murrah breed buffaloes, lactating, multiparous, aged from three to eight years, with a body condition score of 3.5 or higher (0 to 5) and with more than 45 days post partum, were used. These females were randomly distributed into one of the three groups (group 1, group 2 and group 3) with eight subjects in each. On day 0 (day 0), all animals received an ear progesterone implant with 3.0 mg of Norgestomet and an intramuscular (IM) injection with 2.0 mg of estradiol benzoate (EB). The implants were removed after nine days (day 9) and one single dose of PGF2α (0.15 mg d-cloprostenol, IM) was administered to all animals. On the same day, the group´1 and group 3 buffaloes were treated with 500 UI of eCG (IM). Two days later (day 11), 1000UI of hCG (IM) were administered to the group 1 and group 2 buffaloes. After the implant had been removed, an ultrasound evaluation was performed every 12 h, in order to access the ovarian follicular dynamics, using an Aloka 500 equipment with a 5 MHz transrectal probe. Blood samples were also taken on days 0, 9 and 11 to determine the plasmatic concentrations of cortisol and progesterone. No difference was observed in the ovulation rate (group 1: 62.5%, group 2: 50%, group 3: 75%). However, the size of the preovulatory follicles and the plasmatic concentration of cortisol had (P < 0.05). G3 was the most efficient group in promoting the ovulation synchronization, which suggests that this protocol can be used in Fixed Timed Artificial Insemination programs (FTAI) among postpartum, lactating Murrah breed buffaloes’.

## Introduction

The world`s buffalo herd numbers are around 199.28 million, with an annual growth rate of 13.8% (Food and Agriculture Organization - FAO, 2016 ). In Brazil, a population of 1.37 million buffaloes has been reported, making the country one of the largest breeders in Latin America (Instituto Brasileiro de Geografia e Estatística - IBGE, 2016 ).

The inclusion of biotechnologies in the reproductive management of these animals presents an excellent alternative aimed at increasing the reproductive efficiency and, consequently, the productivity and economic inbound of the propriety ( Oba, 2003 ; Ramos, 2003 ).

FTAI is a biotechnique that is widely used and disseminated in bovine herds these days. Its advantages include the following: no need of heat observation; insemination of a larger number of animals in a short window of time; increased number of born calves; concentration of parturition and weaning, leading to more homogeneous feedlots ( Singh and Balhara, 2016 ).

Routine practices in cattle breeding cause expressive stress to the animals, generating discomfort, pain and/or fear. The practices that trigger responses to stress in ruminants include: handling in the corral, insemination, containment and noise ( Maziero *et al*., 2012 ).

With the aim of achieving greater efficiency, different FTAI protocols, generally with three or four managements, are used, with different hormones being applied on various days. However, it is unclear whether the increased frequency of manipulation in the buffaloes` herd that is needed to proceed the hormonal treatments could create a stress and possibly impact the animals fertility.

The aim of the present study was to evaluate the efficiency of three different ovulation synchronization protocols in postpartum Murrah breed lactating buffaloes, verifying cortisol as a stress index to indicate the effect of herd management during the experiment.

## Materials and Methods

### Animals

Murrah breed lactating, multiparous buffaloes, aged from three to eight years, were used. All animals were kept under pasture rotation (Brachiaria sp) with free access to water and mineral salt throughout experiment period.

Twenty-four females were included in the study, all with more than 45 days postpartum, weighting from 450 to 550 kg and with a body condition score(BCS) ≥3.5, ranging from 1 (very poor) to 5 (very fat).

### Experimental design

These females were randomly divided into three experimental groups (group 1, group 2 and group 3), with eight buffaloes in each group. On day 0 (day 0), all animals received a subcutaneous ear implant with 3.0 mg of norgestomet (Crestar®, MSD Animal Health, São Paulo, SP, Brazil) and one IM injection with 2.0 mg of benzoate estradiol (Sincrodiol, Ourofino, Cravinhos, SP, Brazil).

On day 9 (day 9), the ear implant was removed from all animals and 0.15 mg of d-cloprostenol was administered (IM, Croniben, Biogéneses Bagó, Buenos Aires, Argentina). On the same day, the buffaloes from the group 1 and group 3 received one IM injection with 500 UI of eCG (Folligon, MSD Saúde Animal, São Paulo, SP, Brazil). Two days later, the ear implants were removed removal (day 11), and an IM injection of 1000 UI of hCG (Vetecor™, Hertape Calier, Juatuba, MG, Brazil) was administered to the group 1 and group 2 buffaloes.

### Ultrasonography

Transrectal ultrasonographic examinations were performed using an ultrasound equipment (Aloka™ 500) with a linear 5 MHz probe, once daily (every 24 h) on day 9 and day 10, and, twice daily (every 12 h) on day 11, day 12, day 13 and day 14 (6:00 AM and 6:00 PM), to evaluate the follicular dynamics and to determine the time of ovulation. Ovulation was determined by the absence of a dominant follicle, with more than 10 mm in diameter, and its time was measured by the time interval between the last two ultrasound evaluations.

### Blood sampling and hormonal assays

Blood samples were taken by jugular vein punction on days 0, 9 and 11, it was used to dosage the plasmatic concentration of cortisol and progesterone. After collection, the blood samples were centrifuged, and the separated plasma was frozen at -20°C until analyses. The plasmatic concentration of cortisol and progesterone was performed in duplicate using RIA (DPC, Coat-a-countTM, Los AngeIes, CA, USA).

### Statistical analysis

The obtained data expressed in percentages (ovulation rate and ovulation synchronization rate) were statistically analyzed using Fisher`s exact test, and the data expressed in means and standard deviations (SD) were analyzed by ANOVA (follicle diameter, days to ovulation and cortisol plasmatic concentration), followed by Tukey`s test, with all tests having a 5% of significance level.

## Results

### Reproductive status

During the experimental period, plasmatic progesterone concentrations remained below 1 ng/ml on day 0 and day 11, being above only on day 9 due to the presence of the norgestomet implant, indicating that the protocols were used in animals in postpartum anestrus. Within the same days studied (day 0, day 9 and day 11), there was no significant difference between the groups ( Table 1 ).

**Table 1:** Mean ± SEM plasmatic progesterone concentrations (ng/ml) in different experimental groups during hormonal protocols.

	Group 1	Group 2	Group 3	P
Day 0	0.49 ± 0.13^Aa^	0.35 ± 0.07^Aa^	0.34 ± 0.09^Aa^	0.505
Day 9	2.93 ± 0.7^Ba^	2.94 ± 0.12^Ba^	2.91 ± 0.9^Ba^	0.07
Day 11	0.11 ± 0.04^Aa^	0.10 ± 0.04^Aa^	0.10 ± 0.03^Aa^	0.976
P	0.022	0.01	0.038	

Means followed by different uppercase letters in the column and lowercase in the lines, differ by Tukey test at 5% (P < 0.05).

### Follicle development

The mean ± SD of the largest preovulatory follicle diameter (mm) from ovulated buffaloes were: 12.9 ± 2.9 (n = 6); 14.2 ± 1.8 (n = 4); 14.3 ± 1.4 (n = 7), respectively for group 1, group 2 and group 3 (P = 0.47).

When the implant was removed, the mean ± SD of the dominant follicles in the animals that ovulated were: 5.8 ± 2.7 mm (n = 6) for group 1; 8.9 ± 1.6 mm (n = 4) for group 2 and 9.2 ± 1.4 mm (n = 7) for group 3 (P = 0.019). and in the animals, that hadn`t ovulated they were: 5.0 ± 2.1 mm (group 1, n = 2); 6.9 ± 1.6 mm (group 2, n = 4) and 9.0 ± 0 (group 3, n = 1) as shown in Table 1 (P = 0.332), showing that the diameter of the dominant follicle in day 9 varies between the groups, but not between animals that had and had note ovulated when compared in the same group ( Table 2 ).

**Table 2:** Diameter mean ± SEM (mm) of larger follicles in the three experimental groups on ear norgestomet implant removal day (day 9) comparing animals that had ovulated and animals that hadn`t during FTAI protocols.

	Group 1	Group 2	Group 3
Animals that had ovulated	5.8 ± 2.7^Aa^	8.9 ± 1.6^Aab^	9.2 ± 1.4^Ab^
Animals that hadn`t ovulated	5.0 ± 2.1^Aa^	6.9 ± 1.9^Aa^	9.0 ± 0.0^Ab^

Means followed by different uppercase letters in the column and lowercase in the lines, differ by Tukey test at 5% (P < 0.05).

### Ovulation detection

From day 9 to day11, follicular growth rates were observed of 2.8 ± 0.9 mm for group 1; 1.8 ± 0.3 mm for group 2 and 2.2 ± 1.4 mm for group 3 (P = 0.221).

The day of protocol initiation was considered day 0, and ovulation was detected after 12.08 ± 1.36 days (n = 6) in group 1; 12.5 ± 0.71 days (n = 4) in group 2; and, 12.86 ± 0.63 days (n = 7) in group 3 (P = 0.377). There was no difference between groups 1 and 2 regarding the interval between hCG administration and ovulation: 26 ± 13.86 ho (n = 3) for group 1 and 30 ± 16.97 h (n = 4) for group 2 (P = 0.754). The intervals between implant removal and ovulation were 63 ± 30.77 h, 72 ± 16.97 h and 68.57 ± 15.04 h for group 1, group 2 and group 3, respectively (P = 0.812) as shown in Table 3 .

**Table 3:** Mean ± SEM of the interval between the implant removal and ovulation detection in hours.

	Group 1	Group 2	Group 3
Implant removal and ovulation detection interval	63.00 ± 30.77^a^	72.00 ± 16.97^a^	68.57 ± 15.04^a^

Same letters do not statistically significantly differ (P > 0.05).

### Ovulation rates

**Figure 1:**
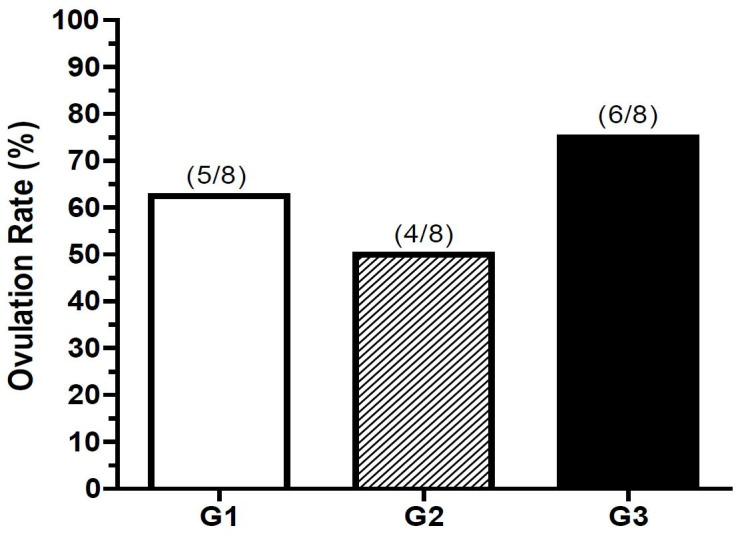
Ovulation rate (%) in each experimental group.

The ovulation rates, shown in Fig. 1 , did not differ among the three experimental groups (P > 0.05) until day 13: 62.5% (5/8) group 1 (eCG + hCG), 50.0% (4/8) group 2 (hCG) and 75% (6/8) group 3 (eCG).

The ovulation synchrony rate, considering animals that had ovulated between days 11 (18:00 PM) and 13 (18:00 PM), were 50% (3/6) for group 1, 100% (4/4) for group 2 and 85.71% (6/7) for group 3 (P > 0.05). The percentages of buffaloes presenting synchrony of ovulation within the interval cited above were: group 1, 37.5% (3/8); group 2, 50% (4/8); and group 3, 75% (6/8), showing no statistical difference (P > 0.05).

### Stress state

The cortisol plasma concentrations (µg/dl) rose during the protocol period ( Table 4 ). There were no statistical differences found in cortisol plasmatic concentrations among the groups. However, cortisol plasmatic concentrations were different within the groups, increasing from day 0 to day 11 in group 1, group 2 and group 3 as follows: day 0<day 9<day 11, (P = 0.014; 0.01 and 0.017).

**Table 4:** Mean ± SEM of the plasmatic concentration of cortisol in the different experimental groups (group 1, group 2 and group 3) evaluated at three distinct times.

	Group 1	Group 2	Group 3
Day 0	0.42 ± 0.05^Aa^	0.48 ± 0.13^Aa^	0.43 ± 0.07^Aa^
Day 9	0.68 ± 0.10 ^ABa^	0.62 ± 0.15^Aa^	0.63 ± 0.09^ABa^
Day 11	0.83 ± 0.11^Ba^	1.22 ± 0.37^Ba^	0.76 ± 0.11^Ba^

Means followed by different uppercase letters in the column and lowercase in the lines, differ by Tukey test at 5% (P < 0.05).

In regard to the mean plasmatic cortisol concentration (μg/dl) of the animals that had ovulated in relation to the animals that had not ovulated, it can be observed that when compared within each group there was no significant difference, but when comparing the total mean of the groups, the animals that had ovulated (0.73 ± 0.08 μg/dl) had a lower plasma cortisol concentration than the animals that had not (1.21 ± 0.30 µg/dl; Table 5 ).

**Table 5:** Mean ± SEM of the plasmatic concentration of cortisol in the different experimental groups (group 1, group 2 and group 3) comparing animals that had ovulated and animals that hadn´t during FTAI protocols.

	Animals that had ovulated	Animals that hadn´t ovulated	P
Group 1	0.75 ± 0.12^a^	0.96 ± 0.20^a^	0.37
Group 2	0.71 ± 0.14^a^	1.7 ± 0.63^a^	0.18
Group 3	0.73 ± 0.14^a^	0.84 ± 0.10^a^	0.7
Total	0.73 ± 0.08^a^	1.21 ± 0.30^b^	0.04

Means followed by different lowercase letters in the lines, differ by Tukey test at 5% (P < 0.05).

## Discussion

The period of the year in which the experiment was carried out was considered favorable to the reproduction of the buffalo species, according to Baruselli *et al.* (2003) ; however, by measuring plasma progesterone levels and the date of the last calving, the animals were presented in postpartum anestrus (P4 < 1ng/ml). The results of plasmatic progesterone concentrations were similar to those found by Presicce *et al.* (2005) who found that progesterone concentrations in lactating females remained below 1 ng/ml up to the next ovulation postpartum.

The higher plasmatic progesterone concentration in day 9 indicates that the use of norgestomet implantation is effective during FTAI protocols in buffaloes. Carvalho *et al.* (2013b) founde similar results when comparing the use of norgestomet implants and progesterone vaginal implants in Murrah buffaloes.

The females that received the ear Norgestomet implant, associated with eCG (group1 and group 3) on the same day as implant removal (day 9), had shown the best ovulation rates and follicular development, with group 3 being the most efficient, probably due to a positive effect of eCG on the pattern of follicular growth ( Baruselli *et al.,* 2004 ; Souza *et al.,* 2009 ).

In previous studies it was evidenced that the administration of eCG in FTAI hormone protocols promotes an increase in FSH and LH, which generates an increase in the diameter of the preovulatory follicle ( Stewart and Allen, 1981 ; Baruselli *et al.,* 2013 ; Carvalho *et al*., 2013a ), mainly by the expression of VEGF wich promotes greater follicular irrigation that supplies the greater nutritional need inherent to the follicular development ( Robinson *et al*., 2009 ; Araujo *et al*., 2011 ), and favors the ovulation rate, allowing greater influx of mediating substances responsible for the degradation of the layers of collagen contained in the tunica albuginea and teak, accompanied by the increase of vascular permeability necessary for follicular rupture ( Jiang *et al*., 2003 ; Siddiqui *et al*., 2010 ). The eCG also favors the development of the corpus luteum because it has a long half-life that allows its connection to the LH receptors of the corpus luteum, promoting greater gene expression of the angiogenic factors VEGF and FGF2 and multiplication of the large luteal cells responsible for the production of progesterone that is essential in maintaining pregnancy ( Souza *et al*., 2009 ; Atli *et al*., 2017 ).

The larger size of preovulatory follicles found in G3, could also be associated with the effect of eCG, wich have similar activity to FSH and LH (Sá Filho *et al.*, 2010), although this influence does not seem to be so intense in group 1, probably due to the administration of hCG as an ovulation inductor on day 11. The group 3 buffaloes had shown potential preovulatory follicles, indicating the efficiency of the wave synchronization protocol. However, differences were not reported among the treatments in the nonovulatory follicles mean diameters. The time from the P4 implant withdrawal to ovulation was similar in both protocols used in the present experiment, and the two protocols did not differ in this respect. The administration of eCG at the moment of the P4 implant withdrawal did not improved the anticipation of ovulation in other studies with buffaloes ( Marques *et al.,* 2003 ; Baruselli *et al.,* 2004 ; Sá Filho *et al.*, 2004; Souza *et al.,* 2009 ). Therefore, this means that the treatments used in the present study had a standard time to ovulation induction.

The results also suggested that the additional support of hCG in the late phase of follicular growth (day 11) in group 1 and group 2 is unnecessary, thereby reducing the cost of the protocol implementation.

In the present study, no differences were observed in cortisol concentrations, but the animals from the group 3 group, which were handled less, had showed a lower increase in the levels during treatments at analyzed times. The group 3 animals had been restrained only twice times for administration of the hormonal protocol, which minimized the stress levels of these buffaloes during the treatment, which was reflected in the ovulation rate, with better reproductive results in this group of females, and better preservation of their well-being. The cortisol concentration increases in this experiment shows that repeated management can cause stress, thereby affecting reproductive performance.

It is known that during FTAI the animals are sensitized, and show a worse temperament on the last day of the protocol, probably due to the management, hormone administration, insertion and removal of the progesterone implant and containment ( Rueda, 2012 ). Thus, the additional management in the group 1 and group 2 for the administration of hCG is likely to have generated stress in the buffaloes leading to reproductive damage that interferes with the ovulation rate, promoting endocrinological disturbances that culminate in decreasing the plasmatic levels of the GnRH, leading to an alteration in liberation frequency and the pulse amplitude of LH, depriving the follicle of the appropriate support of this hormone, and hence reducing the preovulatory follicular diameter and ovulation rate, causing reproductive impairment ( Smith and Dobson, 2002 ; Silva, 2010 ; Garcia, 2013 ).

Fujita *et al*. (2013) confirmed that the hormonal alterations caused by stress could lead to several alterations in the reproductive performance of livestock animals, such as the ovulation rate. Stressful factors may affect not only the follicular development and the ovulation rate but also the oocyte quality which in *in vitro* studies showed failure in embryonic development to through blastocyst stage ( Baruselli *et al*., 2017 ), thereby demonstrating not only the importance of cortisol levels, but also the impact of the number of times that the animals were handled. It was clearly observed that reducing stress during protocols is a key point for future studies applying ovulation synchronization protocols in buffaloes.

In conclusion, the administration of eCG after the removal of an ear implant seems to be favorable for the follicular development, indicating its addition to the synchronization protocol for a more efficient postpartum return to the reproductive activities in lactating Murrah breed buffaloes, and hCG administration on day 11 was not necessary and was considered a stressful agent in buffaloes during the breeding season.
